# The effect of short-term plants cultivation on soil organic/inorganic carbon storage in newly formed soils

**DOI:** 10.1038/s41598-023-45679-y

**Published:** 2023-10-28

**Authors:** Alireza Raheb, Behnam Asgari Lajayer, Venkatramanan Senapathi

**Affiliations:** 1 Department of Soil Science, University of Tehran, Karaj, Iran; 2 Faculty of Agriculture, Dalhousie University, Truro, NS B2N 5E3 Canada; 3 Department of Geology, Alagappa University Karaikudi, Karaikudi, Tamilnadu 630003 India

**Keywords:** Climate sciences, Environmental sciences

## Abstract

Studying total soil carbon (STC), which encompasses organic (SOC) and inorganic carbon (SIC), as well as investigating the influence of soil carbon on other soil properties, is crucial for effective global soil carbon management. This knowledge is invaluable for evaluating carbon sequestration, although its scope is currently limited. Boosting soil carbon sequestration, particularly in arid regions, has direct and indirect implications for achieving over four Sustainable Development Goals: mitigating hunger, extreme poverty, enhancing environmental preservation, and addressing global climate concerns. Research into changes within SOC and SIC across surface and subsurface soils was conducted on aeolian deposits. In this specific case study, two sites sharing similar climates and conditions were chosen as sources of wind-blown sediment parent material. The aim was to discern variations in SOC, SIC, and STC storage in surface and subsurface soils between Sistan and Baluchistan Province (with rapeseed and date orchard cultivation) and Kerman Province (with maize cultivation) in southeastern Iran. The findings highlighted an opposing pattern in SOC and storage concerning soil depth, unlike SIC. The average SOC content was higher in maize cultivation (0.2%) compared to date orchard and rapeseed cultivation (0.11%), attributed to the greater evolution of these arid soils (aridisols) in comparison to the other region (entisols). Conversely, SIC content in the three soil uses demonstrated minimal variation. The mean STC storage was greater in maize cultivation (60.35 Mg ha^−1^) than in date orchard (54.67 Mg ha^−1^) and rapeseed cultivation (53.42 Mg ha^−1^). Within the examined drylands, SIC, originating from aeolian deposits and soil processes, assumes a more prominent role in total carbon storage than SOC, particularly within subsurface soils. Notably, over 90% of total carbon storage exists in the form of inorganic carbon in soils.

## Introduction

Aeolian sediments are wind-deposited materials consisting mainly of sandy or silty particles. Windblown dust, which consists of carbonates, clays, and salts, has been shown to play an important role in the development of desert soils and associated desert soils in arid regions^1^. The southeastern region of Iran is located in an arid region^2^. The 120-day winds of Sistan are considered one of the most important and well-known climatic phenomena that have a great impact on the morphology and environment of eastern and southeastern Iran during the hot season^3,4^. They have various effects on the region. For example, these winds cause dust storms, more evaporation and transpiration, and sand dunes in the region (wind erosion)^3^. Wind erosion destroys soil nutrients that are less than 2 m high, such as humus, clay, and solutes, and transports them to distant areas in the form of dust storms. Dust storms occur in arid and semiarid regions when strong to violent winds stir up large amounts of soil dust and reduce visibility, affecting human health^5–7^. Particles from heavy dust/sand storms typically cover cropland and grassland, causing crop damage and filling rivers and water channels with aeolian material^8^. In recent decades, the importance of the SOC and SIC sources to the global carbon (C) cycle has been largely elucidated^9–17^. Soil carbon is known to be the largest source of C in the terrestrial biosphere, and C changes are one of the most important indicators of the effects of climate on soil formation. Management of soil organic carbon (SOC) requires knowledge of its quantity and effective parameters^18,19^. In recent decades, soil and environmental scientists have conducted extensive studies of soil C. However, the study of various aspects of soil-C is needed in global research programs, especially in arid regions that have recently been affected by global warming. More studies should be conducted to address the problems caused by changes in soil carbon balance in different ecosystems.

This is because the balance of soil carbon in agricultural soils can also affect soil quality improvement, climate change, and crop productivity^20^. Climate and parent material can introduce a range of C contents into ecosystems^21^. Depending on human activities, soils can play an important role as a source or sink of C^22^. In addition, changes in land use and vegetation affect various soil properties, including soil carbon (SOC).

As mentioned earlier, soil carbon (C) is the largest C pool in the terrestrial biosphere and consists of inorganic and organic components. Therefore, it is important and necessary to determine the changes in SOC and soil inorganic carbon (SIC) in different climatic zones to assess the amount of sequestered carbon^19^. Soil organic carbon is one of the most important components involved in global climate change, which is due to its sensitivity to environmental changes^10,23^. Understanding the mechanisms involved in carbon sequestration in soil profiles is critical for assessing regional, continental, and global soil C pools and predicting the consequences of global change^24^.

Knowledge of the spatial variation of SOC is important in drylands as it provides information on soil fertility, water conservation, carbon sequestration, climate change, and the impact of land use practices^25^. Improving organic C content (OC) and promoting C sequestration in arable soils could be important not only for food security and soil health, but also for achieving the Paris Climate Agreement global target of less than 1.5 °C^26^.

In addition to SOC, many soils also contain inorganic C (IC). Soil inorganic carbon stocks (SIC) and their dynamics in arid and semiarid regions covering about one-third of the Earth's surface are very important because the total accumulation rate of SIC is higher than in other biomes^10,27,28^. Lithogenic and pedogenic SIC^29,30^, also play an important role in carbon storage^9^. Several studies have not considered IC as a carbon store^22,31^, probably due to the longer time required for changes in carbonates compared to the shorter time for SOC^32,33^. Most of SIC occurs in arid and semiarid regions and accumulates as carbonate minerals, especially calcite^27^, but the factors affecting the dynamics of SIC are poorly understood^22^.

Understanding the distribution of OC/IC storage in surface and subsurface soils is critical for assessing regional, continental, and global soil C storage and predicting the consequences of global change^16^. However, little is known about OC /IC storage in arid climates, particularly in soils formed on aeolian deposits. On the other hand, with the continuous growth of the world's population, more and more cultivated land is being converted into production land, which currently leads to a degradation of about 33^34^ to 40%^35^ of the world's soils. For this reason, it is very important to study agricultural soils with different uses from an ecological perspective and with an eye on the future. For this study, we selected stable sites without the effects of climate change, topography, parent material, and time in different land uses (agriculture and horticulture) to reduce the effects of other variables on the relationships between SOC and SIC. We investigated the effects of different land uses on SOC, SIC and STC content, storage, and spatial distribution in soils formed on aeolian deposits in the arid regions of southeastern Iran.

## Materials and methods

### Study area and field sampling

**Figure 1:**
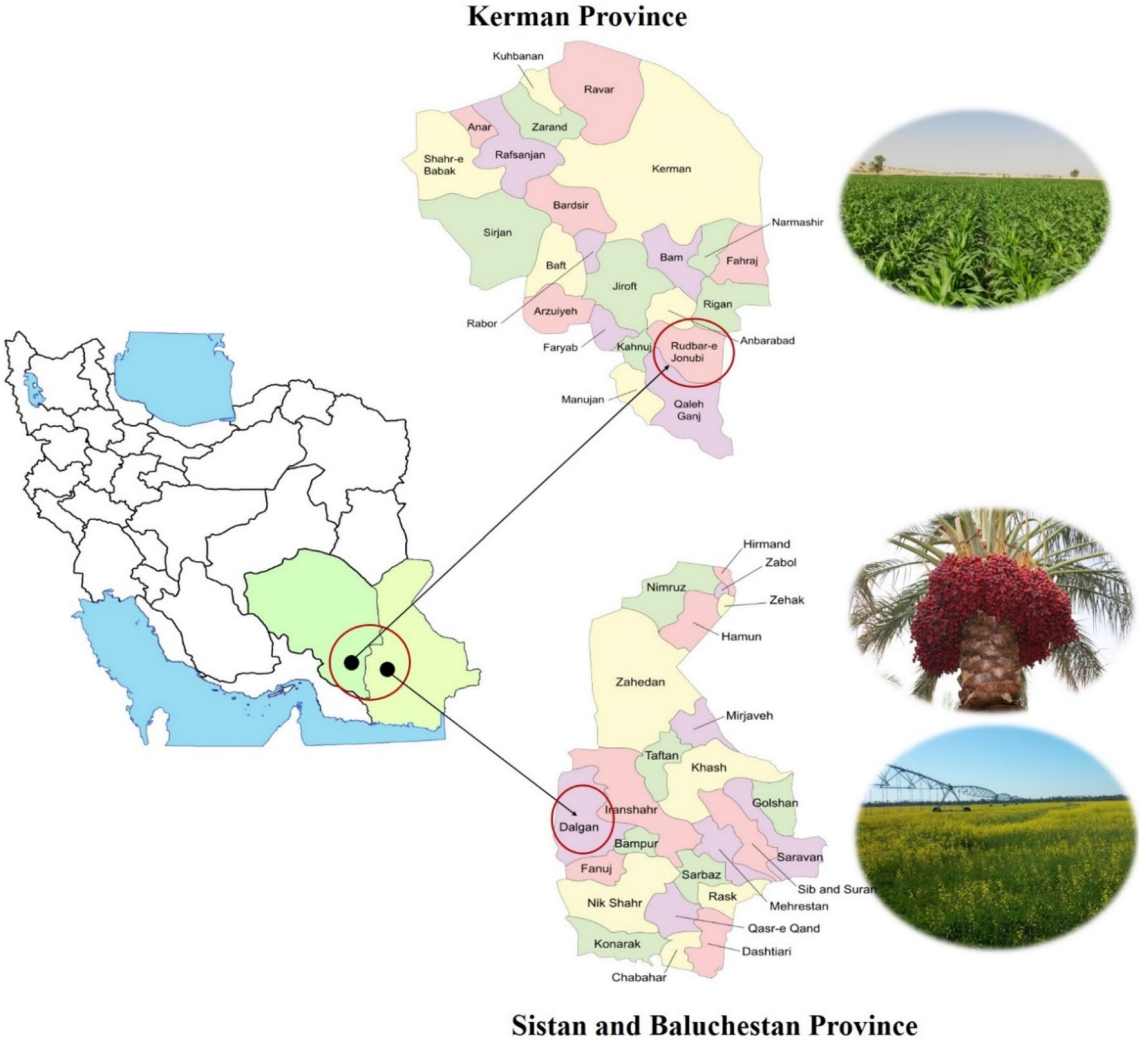
Location of the study area in southeastern Iran showing common land use and cultivation (creating by: paint application version 22h2 windows 10).

The study was conducted in two regions of southeastern Iran (Sistan and Baluchistan province (land use: rapeseed cultivation and date orchard) and Kerman province (land use: maize cultivation) (Fig. 1).

Soil moisture and temperature regimes and evapotranspiration were determined from long-term mean annual precipitation and temperature data using jNSM (java Newhall Simulation Model)^36^ software. The soil moisture and temperature regimes in the studied regions were aridic-hyper thermic (Table 1). The studied regions had shallow geomorphic units and were located about 398–465 m above sea level. The geological features of the studied area have special characteristics, so that the surface of the soil has signs of aeolian deposits, but in the depth of the rounded pebbles indicate alluvial deposits originated in the past from Lake Jazmurian. For this reason, there is evidence of buried soils throughout the area studied. However, in some profiles (especially in Kerman province), this buried layer was not found due to the shallow depth of excavation. The presence of wind deposits caused by the 120-day winds of Sistan on the alluvial deposits, as well as the climatic conditions of the region, slowed down the process of soil development. All sampling sites were located in stable locations with a southeast to northwest orientation and an average slope of less than 2%.

**Table 1:** Geological and climatological properties of the studied regions.

Regions (province)	MAP (mm)	MAT (ºC)	MAET (mm)	SMR	STR	Geological formations
Kerman	156.2	27.4	1620.1	Weak aridic	Hyper thermic	Aeolian deposits/alluvial deposits
Sistan and Baluchistan	91.6	27.5	1498.8	Extreme aridic	Hyper thermic	Aeolian deposits/alluvial deposits

MAP, mean annual precipitation; MAT, mean annual temperature; MAET, mean annual evapotranspiration; SMR, soil moisture regime; STR, soil temperature regime.

### Physico-chemical analyses

**Figure 2:**
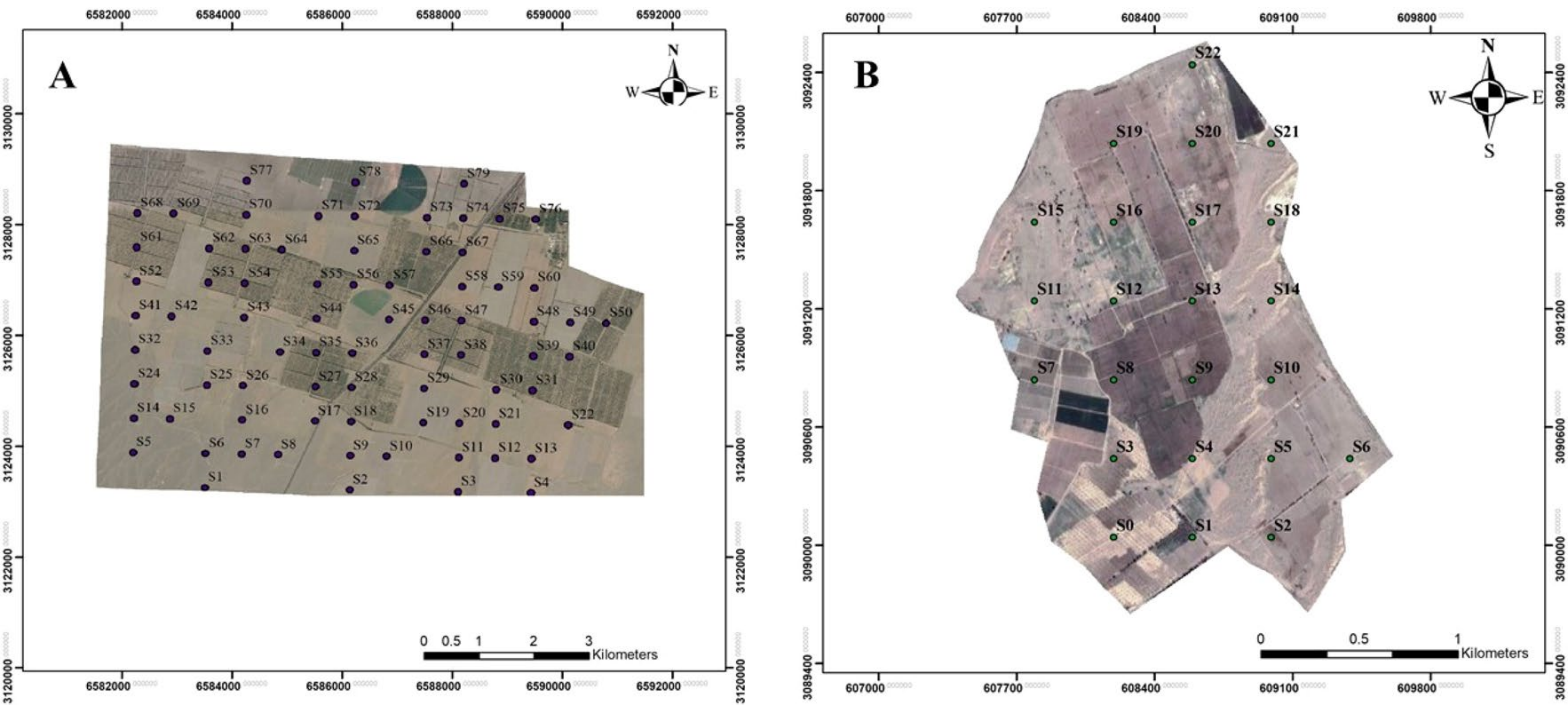
Sampling points positions in both studied regions (creating by: ESRI ArcGIS software version 10.6.1).

18 pedons containing 63 samples from the genetic horizons/layers and 99 surface samples were selected for laboratory analyzes (Fig. 2). After complete analysis of the samples and classification of the soils according to Keys to Soil Taxonomy^37^, four representative pedons from each soil use (maize, rapeseed, and date orchard) with the most distinctive characteristics of the soil taxonomic units were selected for presentation (Table 2). All analyzes were performed on air-dried and sieved (2-mm sieve) soil samples. The percentage of coarse fragments was determined from the weight of fragments with a diameter of > 2 mm/weight of total sample multiplied by 100^38^. Particle size distribution in the fine soil fractions was determined using the hydrometer method^39^. Bulk density was determined by the core method^40^. SOC and SIC (as calcium carbonate equivalent (CCE)) were determined by the Walkley–Black method and the calcimetry method (with increasing reaction time), respectively^41^. STC content was calculated as the sum of SOC and SIC.

**Table 2:** Maximum (Max.), minimum (Min.), means, standard division (S.D.) and standard error (S.E.) of some physicochemical properties surface samples in the studied regions.

	pH	EC (dS m^−1^)	Sand (%)	Silt (%)	Clay (%)	SOC (%)	SIC (%)
Land use: maize cultivation							
Min	8.10	0.37	48.00	4.00	2.00	0.01	8.89
Max	8.90	7.32	94.00	47.00	14.00	1.38	12.80
Mean	8.43	3.47	74.57	18.30	7.13	0.20	10.58
S.D.	0.20	2.25	11.93	9.74	3.31	0.27	0.87
S.E.	0.04	0.47	2.49	2.03	0.69	0.06	0.18
Land use: rapeseed cultivation							
Min	7.90	0.49	67.00	1.00	2.00	0.02	8.40
Max	8.90	13.90	97.00	18.00	15.00	0.23	12.80
Mean	8.51	2.53	86.31	8.44	5.23	0.11	10.28
S.D.	0.25	3.21	6.21	4.14	2.66	0.06	0.91
S.E.	0.04	0.51	0.99	0.66	0.43	0.01	0.15
Land use: date orchard							
Min	8.20	0.41	77.00	2.00	2.00	0.02	8.20
Max	8.90	5.65	95.00	17.00	10.00	0.23	11.50
Mean	8.54	1.47	88.35	7.22	4.43	0.11	10.10
S.D.	0.16	1.29	4.20	3.51	1.82	0.06	0.85
S.E.	0.03	0.21	0.69	0.58	0.30	0.01	0.14

### Calculations and statistical analysis

Equation (1) was used to calculate the amount of total SOC in a pedon with k horizon^16,29^:

1 $$SOC ={\sum }_{i=1}^{k}SOCi=\sum \nolimits_{i=1}^{k}\rho i\times Pi\times Di\times (1-Si)\times {10}^{4}$$

where k is the number of horizons, SOCi is the SOC content (Mg h^−1^), ρi is the bulk density (Mg m^−3^), Pi is the OC content (g C g^−1^) in horizon i, Di is the thickness of the horizon (m), and Si is the volume fraction of fragments > 2 mm. Similarly, SIC was calculated using the Eq. (2):

2 $$SIC ={\sum }_{i=1}^{k}SICi=\sum \nolimits_{i=1}^{k}0.12\times \rho i\times Pi\times Di\times (1-Si)\times {10}^{4}$$

where k is the number of horizons, SICi is the SIC content (Mg h^−1^), ρi is the bulk density (Mg m^−3^), Pi is the IC content (g C g^−1^) in horizon i, Di is the thickness of the horizon (m), and Si is the volume fraction of fragments > 2 mm. The coefficient of 0.12 is the molar fraction of C in CaCO3 to convert the measured carbonates to SIC^42^. The volume fraction of fragments (Si) was calculated for each horizon using the method of the Soil Survey Staff^43^.

To understand the importance of different forms of carbon in deep soils, we also determined SIC, SOC, and STC storage at 0–30, 30–60, and 60–120 cm soil depth based on weighted averages. Descriptive statistical analyzes, including minimum, maximum, mean, and S.E., were performed using SPSS 17.0 software (SPSS, Inc., Chicago, IL).

A factorial design was performed to compare the storage of SOC, SIC, and STC at different depths (0–30, 30–60, 60–120 cm) in soils with different land uses (, date orchard and maize cultivation), with four replicates in homogeneous delineations. Data were analyzed using a mixed linear model and all comparisons were tested using SAS software version 9.4 at a 0.05% significance level.

## Results

### Physico-chemical properties

**Figure 3:**
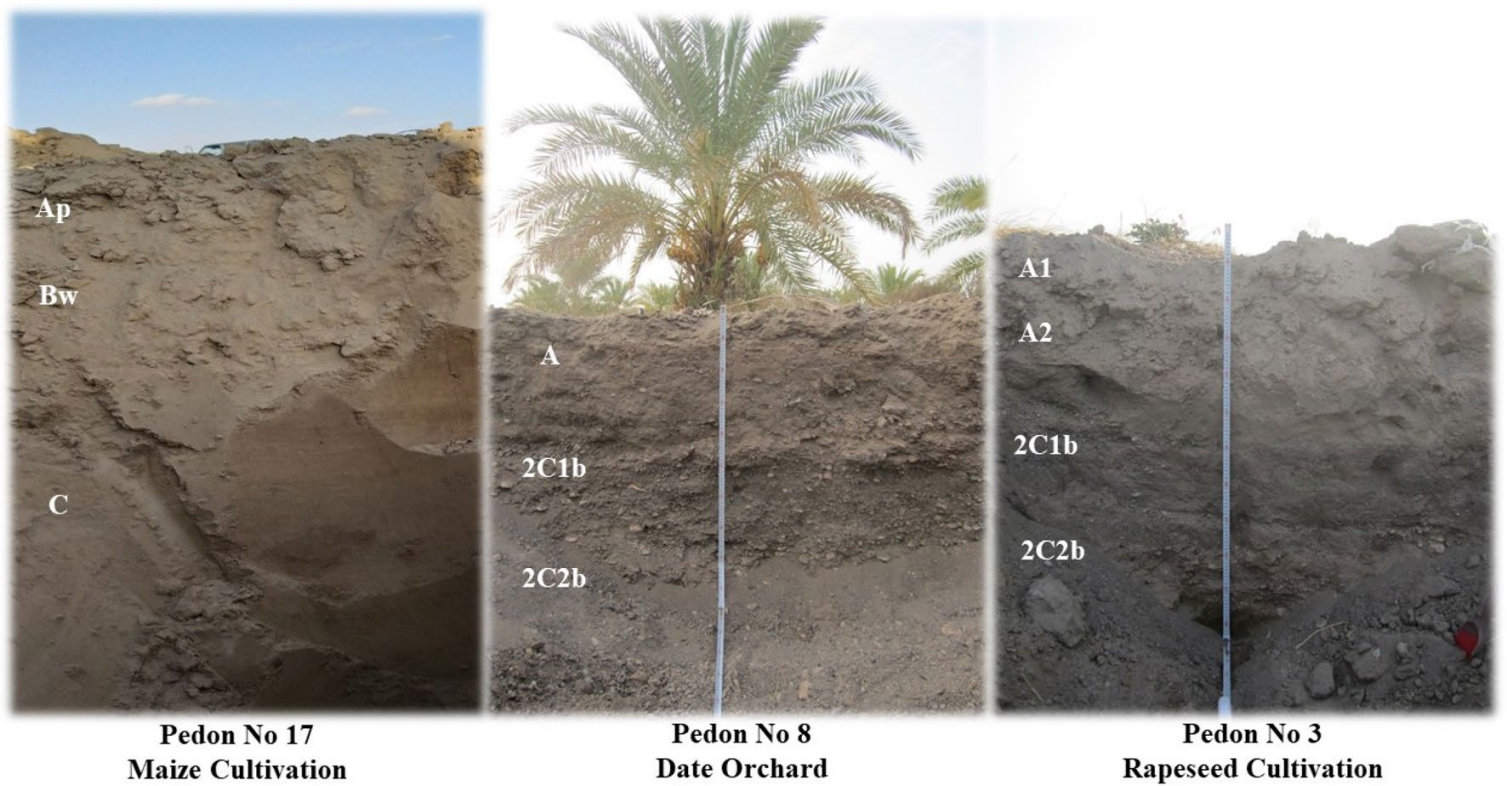
Some of studied pedons with pedogenic horizons in three different land uses.

Figure 3 shows some of the studied pedons with pedogenic horizons in three different land uses. Table 2 shows the classification of the soils, some of the physicochemical properties and the SOC, SIC and STC values for the selected pedons from the different land uses in the studied region. In general, the studied region had less developed soils, including entisols (in rapeseed cultivation and date orchard) and aridisols (in maize cultivation) with shallower soils, higher bulk densities, coarser soil textures, lower SOC, SIC and STC contents and storages (Table 2). The range of bulk densities in rapeseed, maize, and date crops were 1.36–1.57, 1.18–1.56, and 1.34–1.56 g cm^−3^, respectively (Table 2). The results of the profile samples showed that pHe ranged from 8 to 9 in rapeseed cultivation, from 8.0 to 8.7 in corn cultivation, and from 8.2 to 8.9 in date cultivation (Table 2). The electrical conductivity of the saturation extracts (ECe) in rapeseed, maize and date crops were 0.52–9.85, 0.44–5.42 and 0.38–1.56 dS m^−1^, respectively.

### Organic and inorganic carbon contents

SOC and SIC were measured to assess the effects of different land uses on soil formation rates and carbon sequestration. As shown in Table 2, all soil horizons of maize cultivation (in Kerman province) contained higher SOC content (decreasing with increasing depth) than date and rapeseed cultivation in Sistan and Balouchestan province; however, SIC content in the three studied land uses did not show significant changes (Table 2). The highest SIC content (12.8%) was observed in maize and rapeseed cultivation. In the studied region, carbonates were observed only as scattered forms; however, very few visible carbonates were found in these soils, but they were not significant for the diagnosis of pedogenic horizon Bk and calcareous horizon (Table 3).

**Table 3:** Soil classification and properties in selected studied pedons at different land use in studied region.

Horizon	Depth (cm)	CF > 2 mm (%)	B.D (gc m^−3^)	pHe	ECe (dS m^−1^)	SOC (%)	SIC (%)	Sand (%)	Clay (%)	Silt (%)	Tex	SOCs (Mg h^−1^)	SICs (Mg h^−1^)	STCs (Mg h^−1^)
Land use: rapeseed cultivation														
Pedon No. 3—sandy, mixed, hyperthermic, typic torripsamments														
A1	0–14	3	1.51	8.20	0.97	0.06	9.70	94	3	3	S	1.21	23.87	25.08
A2	14–30	5	1.52	9.00	0.53	0.02	9.10	91	5	4	S	0.44	25.23	25.67
2C1b	30–65	6	1.49	8.90	0.52	0.06	10.60	93	4	3	S	2.89	62.35	65.25
2C2b	65–105	9	1.52	8.10	1.59	0.02	9.70	92	5	3	S	1.05	64.40	65.45
Pedon No. 5—coarse-loamy, mixed, subacive, hyperthermic, typic torripsamments														
A	0–13	9	1.42	8.00	9.85	0.10	12.20	80	7	13	L.S	1.65	24.59	26.24
2C1b	13–35	12	1.36	8.00	3.44	0.08	13.10	87	4	9	L.S	2.05	41.39	43.44
2C2b	35–83	25	1.52	8.90	1.05	0.06	13.00	92	3	5	S	3.05	83.58	86.64
Pedon No. 7—sandy, mixed, hyperthermic, typic torripsamments														
A	0–21	3	1.57	8.60	1.14	0.08	10.60	90	5	5	S	2.43	40.68	43.11
2C1b	21–65	5	1.56	8.10	3.25	0.04	10.90	97	2	1	S	2.48	85.29	87.77
2C2b	65–90	6	1.51	8.50	0.87	0.06	8.80	96	2	2	S	2.02	37.47	39.49
Pedon No. 12—sandy, mixed, hyperthermic, typic torripsamments														
A1	0–15	2	1.56	8.00	6.66	0.29	10.00	88	3	9	S	6.63	27.52	34.15
A2	15–31	5	1.51	8.30	1.88	0.10	8.20	88	3	9	S	2.20	22.58	24.79
2C1b	31–65	7	1.54	8.70	0.55	0.06	10.00	96	1	3	S	2.78	58.43	61.21
2C2b	65–121	6	1.56	8.50	0.67	0.08	11.50	94	5	1	S	6.24	113.32	119.56
Land use: date orchard														
Pedon No. 6—coarse-loamy, mixed, subacive, hyperthermic, typic torripsamments														
A	0–45	7	1.34	8.40	1.56	0.21	9.70	79	4	17	L.S	11.78	65.28	77.05
2C1b	45–75	11	1.56	8.20	1.41	0.06	0.70	96	2	2	S	2.37	48.48	50.86
2C2b	75–125	15	1.55	8.40	1.21	0.10	8.20	89	7	4	S	6.32	64.82	71.15
Pedon No. 8—sandy, mixed, hyperthermic, typic torripsamments														
A	0–21	25	1.54	8.50	1.17	0.21	9.40	89	4	7	S	5.09	27.36	32.45
2C1b	21–53	19	1.56	8.60	0.38	0.06	10.00	97	2	1	S	2.30	48.52	50.83
2C2b	53–92	15	1.51	8.70	0.64	0.06	10.00	94	3	3	S	2.85	60.07	62.92
Pedon No. 11—sandy, mixed, hyperthermic, typic torripsamments														
A1	0–21	3	1.34	8.50	1.16	0.12	10.30	87	4	9	L.S	3.14	33.74	36.88
A2	21–28	2	1.38	8.70	0.98	0.08	10.30	77	6	17	L.S	0.72	11.70	12.42
2C1b	28–61	12	1.52	8.40	1.50	0.08	10.30	94	3	4	S	3.35	54.56	57.91
2C2b	61–114	18	1.56	8.60	0.92	0.08	11.80	95	4	1	S	5.15	96.00	101.15
Pedon No. 14—sandy, mixed, hyperthermic, typic torripsamments														
A	0–16	2	1.54	8.80	0.64	0.06	10.90	91	3	6	S	1.38	31.58	32.96
2C1b	16–49	5	1.56	8.90	0.63	0.1	12.10	95	4	1	S	4.69	71.01	75.71
2C2b	49–74	9	1.39	8.90	0.79	0.08	12.10	84	8	8	L.S	2.40	45.92	48.32
2C3b	74–103	13	1.49	8.60	0.74	0.08	11.50	93	4	4	S	2.86	51.88	54.73
Land use: maize cultivation														
Pedon No. 15—sandy, mixed, subactive, hyperthermic, typic haplocambids														
Ap	0–15	2	1.54	8.60	0.44	0.05	10.30	89	4	7	S	1.13	27.98	29.11
Bw	15–46	3	1.48	8.70	0.71	0.03	9.40	92	4	4	S	1.34	50.20	51.54
C	46–95	1	1.56	8.50	0.54	0.02	10.10	91	4	5	S	1.51	91.72	93.23
Pedon No. 16—coarse-loamy, mixed, subactive, hyperthermic, typic haplocambids														
Ap	0–21	4	1.39	8.400	5.42	0.28	10.60	65	11	24	LS	7.85	35.64	43.49
Bw	21–42	7	1.24	8.500	2.95	0.08	12.90	40	20	40	L	1.94	37.49	39.43
C	42–91	2	1.36	8.400	2.56	0.07	12.60	59	12	29	LS	4.57	98.74	103.32
Pedon No. 17—coarse-loamy, mixed, subactive, hyperthermic, typic haplocambids														
Ap	0–27	3	1.42	8.30	4.66	0.18	11.30	67	8	25	LS	6.69	50.43	57.12
Bw	27–49	6	1.26	8.00	3.97	0.04	11.90	57	7	36	LS	1.04	37.21	38.25
C	49–108	3	1.18	8.10	3.99	0.05	12.30	51	9	40	L	3.38	99.68	103.05
Pedon No. 18—sandy, mixed, subactive, hyperthermic, typic haplocambids														
Ap	0–18	5	1.29	8.3	3.64	0.17	10.30	71	10	19	LS	3.75	27.26	31.01
Bw	18–46	3	1.22	8.7	0.98	0.06	10.60	78	5	17	SL	1.99	42.15	44.14
C	46–102	1	1.20	8.4	1.51	0.04	11.00	87	8	5	SL	2.66	87.82	90.48

### Soil carbon stocks

Table 3 presents the calculation of SOC, SIC, and STC reservoirs, derived from their respective contents in relation to horizon thickness. The surface horizons exhibited the highest SOC content (SOC storage) across all regions, while SICs (SIC storage) displayed an opposing trend with larger quantities in subsurface horizons. This SIC trend was similarly reflected in STCs. On the whole, rapeseed showed a greater average total carbon storage from soil surface to subsurface (STCs = 60.35 Mg h^−1^), compared to date orchard (STCs = 54.67 Mg h^−1^) and maize (STCs = 53.42 Mg h^−1^). Table 4 illustrates the outcomes of the SOC, SIC, and STC comparison at specific depths (0–30, 30–60, 60–120 cm) using mixed linear models within the examined regions.

**Table 4:** Maximum (Max.), minimum (Min.), means, standard division (S.D.) and standard error (S.E.) of calculated SOCs, SICs and STCs at different depths (0–30, 30–60, 60–120 cm) in the studied regions.

Depth (cm)	SOC (Mg h^−1^)					SIC (Mg h^−1^)					STC (Mg h^−1^)				
	Min.	Max.	Mean	S.D.	S.E.	Min.	Max.	Mean	S.D.	S.E.	Min.	Max.	Mean	S.D.	S.E.
Land use: rapeseed cultivation															
0–30	0.82	9.59	4.48ab	2.42	0.39	44.81	63.00	52.06b	4.26	0.68	46.31	67.22	56.54c	4.88	0.78
30–60	2.48	2.89	2.76b	0.19	0.10	58.43	85.29	70.66b	12.48	6.24	43.44	87.77	64.42c	18.22	9.11
60–120	1.44	7.95	4.83ab	2.86	1.43	68.69	218.03	122.15a	66.48	33.24	72.39	225.98	126.98ab	68.81	34.40
Land use: date orchard															
0–30	0.83	9.94	4.81ab	2.48	0.41	40.80	60.23	51.48b	4.95	0.81	44.31	64.55	56.29c	5.04	0.83
30–60	2.43	7.07	4.18ab	2.02	1.01	51.21	61.81	56.11b	4.45	2.23	53.64	65.66	60.29c	5.54	2.77
60–120	2.75	5.34	4.64ab	1.27	0.63	50.49	112.63	79.96b	29.23	14.61	53.24	117.97	84.61bc	30.08	15.04
Land use: maize cultivation															
0–30	0.43	52.06	7.82a	10.01	2.09	40.44	57.21	49.65b	4.47	0.93	44.61	108.20	57.47c	12.00	2.50
30–60	1.42	3.53	2.29b	0.90	0.45	60.12	74.24	66.85b	6.29	3.15	62.02	77.77	69.14c	6.83	3.42
60–120	2.58	8.85	4.86ab	2.75	1.37	124.60	191.11	149.60a	31.56	15.78	128.82	199.96	154.46a	33.62	16.81

## Discussion

### Vertical distribution of soil carbon in the pedons

The low levels of SOC and SIC observed in the examined region can be attributed to reduced chemical weathering, biomass growth, and residue production. The arid climate significantly limits biomass generation, leading to the lowest SOC levels within this area. According to Evans et al.^44^, precipitation was found to positively correlate with SOC levels, whereas temperature showed a negative correlation in Inner Mongolia. This pattern could be explained by the accelerated production of SOC relative to decomposition with higher precipitation, and conversely, faster decomposition compared to production with higher temperatures. As depth increases, the quantity of OC in profiles diminishes, while IC exhibits irregular fluctuations. In the context of sustainable agriculture, emphasis is placed on soil quality and organic matter (OM), with plant residues representing a vital source of soil OM. These residues play a crucial role in mitigating climate change by capturing carbon, thereby offsetting carbon dioxide emissions and other greenhouse gases^45^. As shown in Table 2, all soil horizons of maize cultivation contained higher SOC contents than those of date and rapeseed cultivation. Maize is an annual and important crop in Iran, which is rotated with crops such as wheat and rapeseed. Each year, large amounts of crop residue are removed from Iranian corn fields, preventing a large amount from OM being returned to the soil and decreasing soil fertility. Maize has a spreading root that spreads from all sides and absorbs the necessary nutrients from the plant. Corn root development is highly dependent on soil texture or fertility and penetrates to a depth of 50 cm. The speed of spreading and development of the maize root is high and it develops up to 60 cm around the stem within four weeks. For this reason, it is logical that the average SOC content in maize cultivation is higher than in the other two types of use.

Plant residues play the most important role in OM production and soil productivity^46^.

The results of Mirzavand^47^ showed that maize residues led to an improvement in soil OM such that conservation of residues at the surface (0–10 cm depth) caused a 2% increase at OM and a 1% increase at depth. Heidari^48^ also reported that corn residues increased SOC content by 25% compared to wheat residues. The effect of cultivation on the next crop in the rotation depends on several factors, such as crop type, length of growing season, soil moisture, plowing method, irrigation method, use of nitrogen fertilizer, and the amount and quality of crop residue returned to the soil^49^.

Maize, rapeseed, and dates stand out as some of the most commonly consumed crops for sustenance and deriving nourishment from the soil. The cultivation of these crops in the southern and southeastern regions of Iran takes advantage of the soil's light texture and helps mitigate wind erosion, ultimately resulting in increased food production from the land. However, it's important to note that the deficiency of organic carbon (OC) within the soil may stem from several factors, including excessive utilization of chemical fertilizers, the failure to reintegrate plant residues into the local soil, and even their combustion, all of which contribute to a decrease in soil organic matter (OM).Chemical results show that the amount of calcium carbonate increases with depth in all three regions, while the content of SIC does not show significant changes in the three land uses studied. Signs of secondary calcium carbonate accumulation were not detected in the studied profiles, and wind deposits transferred from other sites cause the presence of IC in the samples.

### Soil carbon storages

For each pedon, the highest OM content was observed in the surface soil horizons (A horizons). Table 3 shows the total OM storage SOC, SIC and STC in each pedon. The lowest amounts of SOC (average: 2.65 Mg h^−1^) were determined in the rapeseed crop due to poor vegetation cover, few root residues, and lower OC input to the soil. Fast-growing crops such as rapeseed decompose quickly when returned to the soil and therefore have little effect on increasing SOC content^50^. The results of Lupwayi et al.^51^ showed that rotation of rapeseed with field crops increased SOC content and soil microbial community activity. On the other hand, the highest amount of SOC (average 3.88 Mg h^−1^) was associated with date orchard, as it was influenced by proper human management and fertilized with animal manure in recent years to increase fertility and crop yield.

The average SIC values displayed variations of 57.19 Mg h^−1^, 50.78 Mg h^−1^, and 50.76 Mg h^−1^ for maize, date, and rapeseed, respectively (Table 3). The region where maize is cultivated exhibited notably higher root-based respiration rates compared to other areas, underscoring their significance in the generation of inorganic carbon (IC) in this specific zone. This phenomenon was associated with a remarkable increase in carbonate dissolution by a factor of 5 to 10, as roots actively contributed to the process^52^. Notably, the solubility of carbonates adjacent to roots elevated due to the augmented CO_2_ concentration within the rhizosphere in contrast to the atmosphere (sometimes by up to 100 times), along with a lower local pH^53^.

### Importance of carbon storage in subsurface soil (> 60 cm)

**Figure 4:**
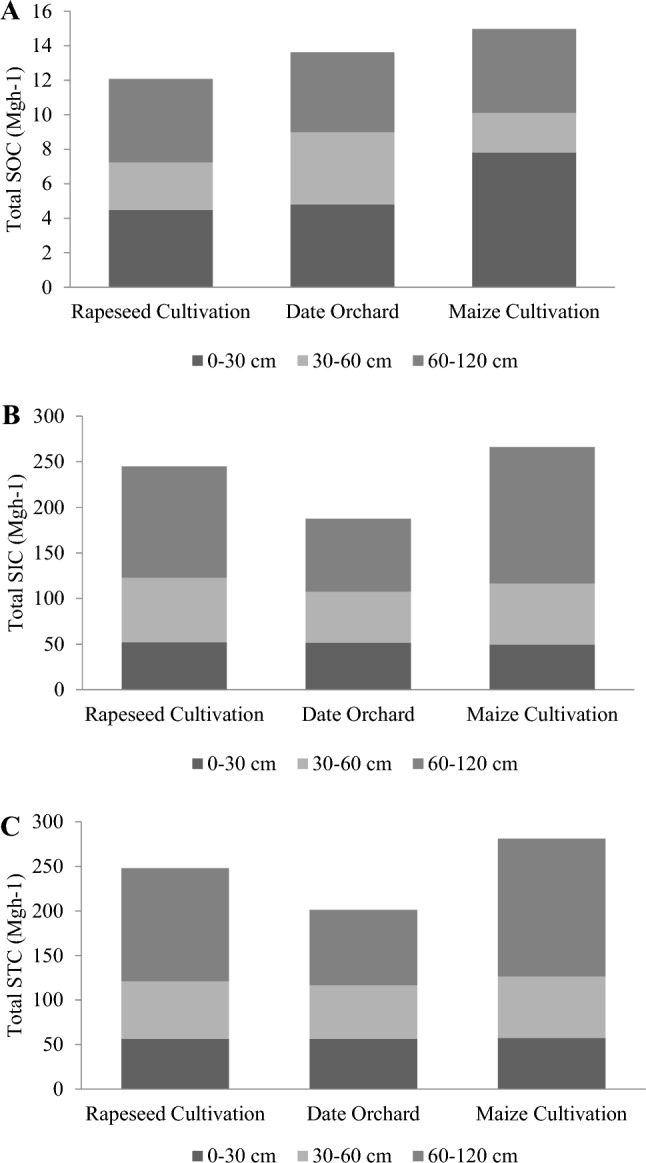
Storage of soil C forms as a function of depth (0–30, 30–60, 60–120 cm) in the studied regions, (**A**) SOC, (**B**) SIC, and (**C**) STC.

Although it is known that subsurface horizons are important for soil carbon storage, most studies have focused on carbon storage in the upper soil horizons^17,24^. Some studies have indicated that soil carbon stocks are greatly underestimated if the amounts stored in the subsurface are not taken into account^42,54^. Table 4 shows the storage values of organic, inorganic, and total carbon at three different depths of 0–30, 30–60, and 60–120 cm. The results show SOC storage at different soil depths, indicating that relatively large amounts of SOC are stored in subsurface layers. These amounts accounted for about 40%, 32%, and 34% of SOC in rapeseed, maize, and date orchard, respectively (Fig. 4).

In contrast, the results of this work showed that SIC storage in the deeper soils is also very important and the highest percentage of SIC (more than 43%) was observed in the depths of 60–120 cm for all land uses. Also, more than 43% of the total carbon storage (SOC + SIC) in the studied soils occurred in the depths of 60–120 cm. Thus, our results are in agreement with the studies of Raheb et al.^13^, Wang et al.^16^, and Jobbágy and Jackson^55^.

## Conclusion

The region has faced formidable challenges in planning agricultural development projects and maintaining crop yields due to recent droughts, scarce water resources, inadequate irrigation efficiency, declining soil fertility, acute scarcity of SOC and nutrients for sustainable production, and the inability to meet water demands for cultivation. Upholding or increasing soil organic matter (OM), particularly in arid areas (due to high temperatures) and sandy soils (due to abundant aeration), remains challenging due to elevated decomposition rates. The influx of wind-driven sediments in recent times has hindered soil development in the study area, rendering soils relatively young and underdeveloped. Low levels of SOC and SIC in the region stem from diminished chemical weathering, biomass growth, and residue production. Consequently, soil formation processes have been limited, resulting in reduced soil carbon content. Unlike SIC contents, SOC stocks exhibited an inverse relationship with increasing soil depth. In the maize-growing zones, the mean STC storage (STCs) was higher (60.35 Mg h^−1^) compared to date orchards (54.67 Mg h^−1^) and rapeseed areas (53.42 Mg h^−1^). Additionally, over 43% of STC storage in the studied regions was located in deep soils (> 60 cm). Among the three land use types, soil inorganic carbon constituted the majority (> 90%) of STC, indicating that in dry and underdeveloped soils, IC predominates, while conditions are less favorable for OC formation.

In general, crop residues, particularly those from rapeseed and maize, bolster microbial activity during the growing season in arid regions. They achieve this by mitigating surface water evaporation, enhancing soil moisture, optimizing soil temperature conditions, and promoting root growth. These effects culminate in improved soil physical and chemical attributes, especially OC levels. In essence, soil carbon content hinges on the equilibrium between organic matter inputs and losses (through decomposition and erosion). When losses escalate while inputs remain constant, soil OM diminishes. Conversely, when losses stabilize and inputs increase, soil OM rises (see Table 4). In conclusion, studies in the selected regions have proposed strategies for sustainable development and management in drylands, aiming to alleviate constraints and optimize carbon resource utilization. These strategies, transferrable to similar regions worldwide, encompass tactics such as curbing wind erosion (soil conservation), implementing appropriate crop rotation, utilizing available organic fertilizers in arid zones, optimizing water resource management, and adopting modern irrigation systems.

## Data Availability

The data used to support the findings of this study are available from the corresponding author upon a reasonable request.
